# A Two-Dimensional Position and Motion Monitoring System for Preterm Infants Using a Fiber-Optic Pressure-Sensitive Mattress

**DOI:** 10.3390/s25154774

**Published:** 2025-08-03

**Authors:** Giulia Palladino, Zheng Peng, Deedee Kommers, Henrie van den Boom, Oded Raz, Xi Long, Peter Andriessen, Hendrik Niemarkt, Carola van Pul

**Affiliations:** 1 Department of Electrical Engineering, Eindhoven University of Technology, 5612 AZ Eindhoven, The Netherlands; z.peng@tue.nl (Z.P.); h.vandenboom@luxisens.com (H.v.d.B.); o.raz@tue.nl (O.R.); x.long@tue.nl (X.L.); p.andriessen@mmc.nl (P.A.); C.vanPul@mmc.nl (C.v.P.); 2 Department of Clinical Physics, Maxima Medical Center, 5504 DB Veldhoven, The Netherlands; 3 Department of Neonatology, Maxima Medical Center, 5504 DB Veldhoven, The Netherlands; deedee.kommers@mmc.nl (D.K.); hendrik.niemarkt@mmc.nl (H.N.); 4 Luxisens Technologies B.V., 5612 AZ Eindhoven, The Netherlands

**Keywords:** motion monitoring, position monitoring, premature infants, fiber-optic mattress, smart mattress, test protocol

## Abstract

Monitoring position and movements of preterm infants is important to ensure their well-being and optimal development. This study evaluates the feasibility of a pressure-sensitive fiber-optic mattress (FM), made entirely of plastic, for two-dimensional analysis of preterm infant movements and positioning. Before clinical use, we developed a simple, replicable, and cost-effective test protocol to simulate infant movements and positions, enabling early identification of technical limitations. Using data from 20 preterm infants, we assessed the FM’s potential to monitor posture and limb motion. FM-derived pressure patterns were compared with camera-based manual annotations to distinguish between different positions and out-of-bed moments, as well as limb-specific movements. Bench-test results demonstrated the FM’s sensitivity to motion and pressure changes, supporting its use in preclinical validation. Clinical data confirmed the FM’s reliability in identifying infant positions and movement patterns, showing an accuracy comparable to camera annotations. However, limitations such as calibration, sensitivity to ambient light, and edge-related artifacts were noted, indicating areas for improvement. In conclusion, the test protocol proved effective for early-stage evaluation of smart mattress technologies. The FM showed promising clinical feasibility for non-obtrusive monitoring of preterm infants, though further optimization is needed for robust performance in neonatal care.

## 1. Introduction

Each year, more than 15 million infants are born preterm, defined as birth below a gestational age of 37 weeks. These preterm infants face increased risk for mortality and persistent health problems that can endure throughout their life [1]. In addition, these babies often need to be admitted to medical facilities and require close monitoring of clinical conditions and vital signs.

In addition to standard monitoring practices, visual evaluations of infant movement patterns and position play a crucial role in examining their clinical conditions and sleep behavior, as decreased movement can be indicative of health problems, such as hypoglycemia and sepsis [2], and position is associated with different clinical characteristics, such as breathing pattern and feeding tolerance, and can be a sign of disease [3,4,5].

The movements and positioning of preterm infants are currently evaluated via direct visual observations by caregivers or medical personnel, which are inherently subjective and limited in duration, particularly as many neonatal intensive care units (NICUs) use nowadays single room care. To address the need for continuous, quantitative, and objective monitoring, innovative technologies and methods are being investigated to facilitate more accurate tracking [6,7].

Position monitoring until now mainly uses camera methods, often coupled with machine learning for analysis. Jahn et al. [8] evaluated pose estimation models for infant movements in the General Movement Assessment (GMA). In particular, the study highlighted the importance of dataset-specific retraining and optimal camera positioning in infant pose analysis.

Motion information can be derived from commonly used sensors, such as photoplethysmography (PPG) [9] or electrocardiography (ECG) [10]. However, these methods are designed to be motion robust, and the use of embedded filters can restrict their efficacy. Moreover, they do not provide any position information. Non-contact approaches, especially those that apply video technology [11,12], provide significant advantages in the analysis and characterization of infant behaviors.

Wearable sensors can be also used in posture monitoring. Airaksinen et al. [13] introduced a smart wearable jumpsuit with limb-placed inertial sensors to monitor infant posture and movement. In a study on 22 typical infants, it captured motion data and used a custom annotation system to classify postures and movements. A convolutional neural network (CNN) trained on these data reached human-level accuracy, outperforming traditional machine learning methods, and was able to distinguish between high- and low-motor-performance infants, showcasing its potential for early neurodevelopmental impairment detection in a scalable and non-intrusive manner.

However, these approaches encompass several limitations, including the potential for camera occlusions, dependence on lighting conditions, privacy issues, and the requirement for labor-intensive data analysis.

Various forms of smart mattresses have been introduced in motion monitoring. For example, the study conducted by Joshi et al. [14] explored the use of an electromechanical film sensor (EMFi) mattress designed for infants for motion detection. This investigation demonstrated a high level of effectiveness, with an area under the curve (AUC) value of 0.90. However, the EMFi turned out to be particularly susceptible to noise and artifacts, and the system is able to provide only a one-dimensional output, lacking information about the infant’s position. Aziz et al. [15] evaluated a two-dimensional approach, which exhibited exceptional precision in identifying patient movements, achieving an AUC of 0.97. However, it is imperative that further refinements and validation are undertaken before this technology can be used effectively in real-time applications.

In our previous studies [16,17], we used a two-dimensional mat designed using fiber optic sensors, which is able to identify alterations in pressure. These changes are discerned on the basis of the phenomenon of light coupling that occurs between intersecting fibers. The performance of the mattress achieved an AUC of 0.91 compared to manual annotations derived from video recordings. Moreover, we showed that mat-based motion detection was synchronized to the ECG-based motion [18].

However, to further develop the FM for clinical practice, a rigourous testing procedure is needed, as legislative frameworks, such the Medical Device Regulation in Europe [19], require that medical devices undergo a thorough testing, ensuring that innovative technologies are safe and effective for clinical care [20].

In this study, we present a simple, replicable, and affordable testing protocol tailored for the bench-test phase of the smart mattresses. Such a protocol must be in place before initiating any clinical evaluations on human subjects. Moreover, we aim to investigate the feasibility of the FM in detecting position and movement patterns of infants. The two-dimensional framework of the FM system enables both comprehensive monitoring of the infant position, including instances when the infant is out of bed, as well as precise limb movements.

## 2. Materials and Methods

### 2.1. Fiber-Optic Pressure-Sensitive Mattress

**Figure 1:**
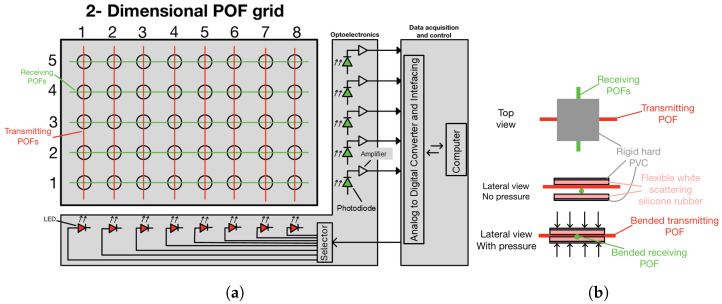
Electronic schema of a 8 × 5 fiber-optic pressure-sensitive mattress. (**a**) Schematic view of the electronic system of the FM. (**b**) Schematic view of a POF crossing.

The fiber optic pressure-sensitive mattress (FM) functions as a two-dimensional pressure detection system composed of a polymer optical fiber (POF) grid [17]. Figure 1a provides a schematic overview. The design features two sets of POFs arranged in perpendicular orientations to form the grid structure. In this setup, fibers aligned in one direction are linked to light-emitting diodes (LEDs), whereas those aligned perpendicularly are connected to photodiodes, forming the optoelectronic unit. Each intersection in the grid acts as a different pressure sensor, and the number of pressure sensors can be easily varied. This configuration enables the coupling of light from the LED-connected POFs to the photodiode-connected ones, if pressure is applied on the sensor, generating a pressure signal over time. The data acquisition and control module (DAC) is tasked with managing the LED transmitters and processing the information received from the photodiodes. Furthermore, the DAC controls the selector to individually activate each LED during the column-by-column scanning process. Consequently, the DAC constructs the two-dimensional pressure profile by simultaneously recording the outputs from the photodetectors on a line-by-line basis.

As mentioned above, each POF intersection forms a pressure sensor, which can be indicated using its coordinates as (r, c), where r is the row number and c the column. Figure 1b illustrates a schematic representation of a POF intersection. The two POFs are incorporated into a compact patch composed of a thin, flexible, light-scattering material, such as white silicone rubber; and a thin, rigid material, such as hard PVC. When a force is exerted on the intersection, the scattering material slightly bends the fibers smoothly. This results in two forms of bending: a micro-bending due to the contact between the fibers and a macro-bending due to the slight bending of the fibers. This process allows for a small portion of light to be emitted from the transmitting fiber, with the intensity of the emitted light being proportional to the applied force. The scattering material subsequently diffuses the light in multiple directions, with a portion being coupled into the receiving fiber. Additionally, the flexible material plays a crucial role in enhancing the FM’s durability, as it provides the fibers with an appropriate bending radius at the intersection when pressure is applied, and it partially absorbs forces away from the fiber crossing.

The FM’s durability has been tested with a test setup in an extreme situation where a weight of 1 kg was dropped at a single POF sensor crossing with a frequency of 30 times per minute for 1 h, and no permanent damage was observed. However, further investigation is necessary for better quantification. Moreover, the plastic materials used to design and build the FM make it particularly suitable for use in clinical settings, like NICUs, where the cleaning and disinfection process is a crucial step to ensure the well-being of the patients. Of course, refinement of the prototype sensor towards a clinical sensor for neonatal applications will also include investigating better-suited materials for the humid and warm NICU incubator setting.

### 2.2. Device Testing

**Figure 2:**
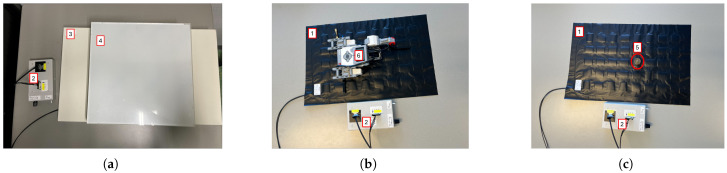
Setup and materials for each test. For a clear view of the materials and the FM, the regular mattress is not shown in the pictures. (1) FM, (2) controller: (**a**) Linearity test: final configuration of the plates on the FM without the regular mattress. (3) 2.7 kg plate, (4) 1.5 kg plate: (**b**) Motion sensitivity and reproducibility tests: positioning of the Lego Mindstorm robot on the FM without the regular mattress. (6) Lego Mindstorm robot: (**c**) Precision test: example of the position of the calibration weight on one node of the FM. (5) Calibration weight.

The FM device must undergo rigorous testing before entering a clinical study to focus on examining its characteristics and assessing its accuracy in sensing the pressure exerted on its sensors. Therefore, we developed a low cost and easy protocol to evaluate the FM and its future versions. This protocol consists of a series of tests, each designed to analyze a specific feature of the device. To more accurately replicate an NICU/NMCU setting, a standard mattress has been used when suitable. The tests introduced are the following:

a Drift: To study the stability of the FM over a long period of time. In this test, the FM was empty, and it was turned on for an extended period of time (8 h and 72 h).
b Linearity: To address the linear response of the device to the applied linear pressure. The materials and the test configuration are shown in Figure 2a. The test was performed using the FM, a regular mattress used in neonatal care, and six plastic plates (one of 2.7 kg and five of 1.5 kg each), covering the entire surface of the sensor mat of 60 × 32 cm^2^. The test was conducted as follows: Initially, the FM was left for 60 s with just the mattress placed above it. Subsequently, the first plate (2.7 kg) was positioned on top and left for another 60 s. This process was repeated by adding additional plates at 60 s intervals until all plates were in place. Post-placement of the final plate, the test proceeded for an additional 60 s.
c Reproducibility: To assess the ability of the FM to sense the same information without changing the input. The materials and the test configuration are shown in Figure 2b. The test was carried out using the Lego Mindstorms robot (weighing 512.4 g, representing an infant born at 24 weeks of gestation). The test was conducted as follows: Initially, the FM was left undisturbed for 60 s, with solely the mattress positioned on top. Subsequently, the Lego robot was centrally placed for a duration of 60 s, remaining immobile throughout this interval. The Lego robot was then removed, leaving the FM unoccupied for another 60 s. This sequence of steps was iterated four times.
d Motion sensitivity: To test the possibility to sense small movements. The materials and the test configuration are shown in Figure 2b. The test was carried out using the Lego Mindstorms robot previously mentioned, which is able to mimic the movements of an infant lying on the FM, as it is programmed to move constantly with the right arm, leg, and left arm alternately. The test was conducted as follows: Initially, the FM was left undisturbed for 60 s with solely the mattress positioned on top. Subsequently, the Lego robot was centrally placed for a duration of 60 s, remaining active with the motion pattern throughout this interval. The Lego robot was then removed, leaving the FM unoccupied for another 60 s. This sequence of steps was iterated five times, with the robot repositioned in the same location each time. The sequence of movements of the robot is shown below. 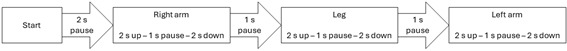
e Precision: To understand if all the FM nodes respond in the same way when the same pressure is applied on each one individually and to understand if mechanical cross-talk is present. The materials and the test configuration are shown in Figure 2c. The results should show that one node is able to sense the pressure only when the weight is applied on it; also, the surrounding nodes should not sense pressure changes. The test was conducted as follows: Initially, the FM was left unoccupied for a duration of 60 s. Subsequently, a calibration weight (100 g) was placed at sensor (1,1) for 10 s. The weight was subsequently shifted along the grid, progressing row by row until it reached sensor (8,5), with the weight being stationed on each node for 10 s. After the 10 s at sensor (8,5), the weight was removed, and the FM remained vacant for another 60 s.

### 2.3. Clinical Study

The FM was employed for a first clinical study in 2022 for a early one-dimensional analysis of movements. This study comprised 20 preterm infants admitted to the NICU or NMCU of Maxima Medical Center, Veldhoven, The Netherlands. The infants had a median gestational age of 29.5 weeks (range: 25.3–36.1 weeks), a postmenstrual age of 33.3 weeks (range: 29.3–37.9 weeks), and a median study weight of 1615 g (range: 960–2725 g).

After the parents of the infants signed the written parental consent, the infant was placed on the standard mattress within the incubator or open bed, and the FM was placed underneath it, without being in direct contact with the patient. In this study, an FM with a configuration of eight transmitting and five receiving fibers was used, for a total of 40 sensors. Measurements were performed during the day for a duration of 2–5 h, during which routine daily care was provided to all participants.

Also, chest impedance (CI) and electrocardiogram (ECG) signals were collected as part of routine clinical monitoring using a neonatal patient monitor (Philips IntelliVue MX 800, Philips, Böblingen, Germany) and stored in a data warehouse (Philips PIIC iX, Data Warehouse Connect, Philips, Andover, MA, USA).

During the test, a camera system (UI-3860LE-C-HQ, resolution 1280 × 720 pixels, and frame rate of 10 fps) was set up to record the upper body and head of the infant.

**Figure 3:**
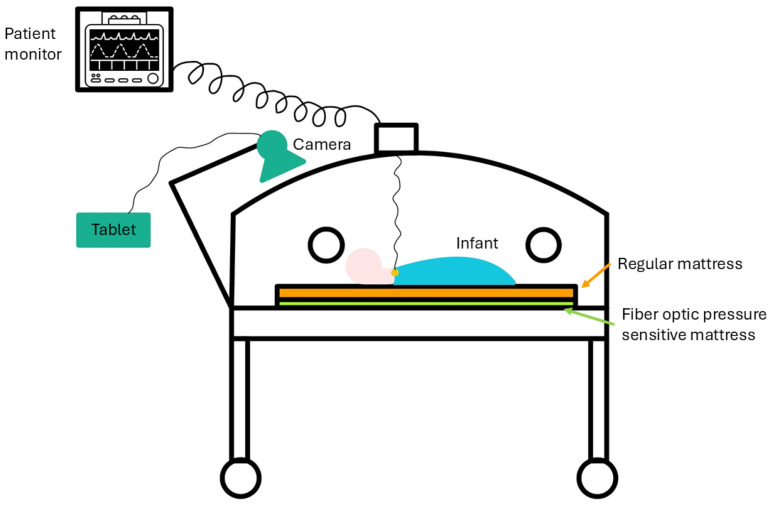
Schematic view of the clinical study setup.

The setup of the study is shown in Figure 3.

The synchronization between the camera and the FM was performed manually using simultaneous start moments at the beginning of each measurement. The synchronization between the FM and the ECG / CI was carried out by briefly disconnecting the ECG for a short period of time (<60 s). For the first three infants, this synchronization was performed by pressing a sensor on the FM at the beginning of the test.

### 2.4. Signal Processing

#### 2.4.1. Processing of Device-Testing Data

**Figure 4:**
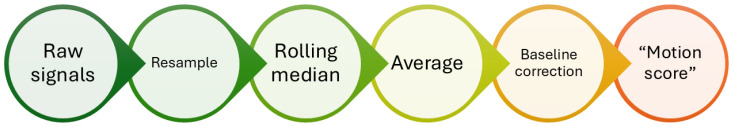
Signal-processing pipeline of testing data.

The data obtained from the tests were analyzed as shown in Figure 4. Initially, the raw data were interpolated to ensure correct sampling at 50 Hz. Subsequently, a moving-window median filter was applied to decrease noise, using a 3 s window with a step of 1 sample (0.02 s). The signals were then averaged to produce a single final signal, to which a baseline correction was applied by subtracting the minimum value. In this way, a signal, named motion score, was derived per test from the 40 sensors.

The data of the precision test were only interpolated to ensure a sampling frequency of 50 Hz, and the results were generated from the raw pressure signals.

The evaluation of the test results was made by comparing them with the expected results in the ideal cases or with a visual inspection of the results. For the drift data, the percentage of drift each hour and in each 24 h period was calculated, and the median values were retrieved.

#### 2.4.2. Processing of Clinical Study Data

#### Video Recordings

An uninterrupted one-hour segment of the video recordings was selected and manually analyzed using Matlab’s Video Labeler Tool. The video was segmented into consecutive non-overlapping epochs of 10 s. Each epoch was classified into one of the following categories: still, gross movement (characterized by torso or chest activity), or fine movement (comprising isolated actions of the head, hands, arms, fingers, or facial expressions). If these three main categories were not identified, further classification was performed. During the same time, additional annotations were applied for intervention, camera movement, and inappropriate viewing angles along with the main categories. Subcategories were also used to detail specific movements of the limbs and heads when visible. In addition, the posture of each infant was annotated for the available recordings at the start of each one.

#### Fiber-Optic Pressure-Sensitive Mattress Data

**Figure 5:**
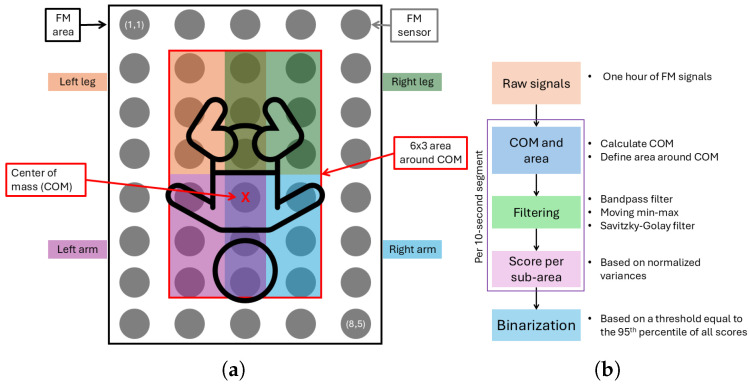
Motion monitoring using the fiber-optic pressure-sensitive mattress. (**a**) Non-edge case of COM, area around COM and sub-areas definitions. Orange = left leg, green = right leg, purple = left arm, blue = right arm. (**b**) Signal processing pipeline for each 10 s segment.

From the FM data collected during the clinical study, only the annotated one-hour segment was used for analysis. To ensure data quality for motion analysis, we excluded any segments that involved interventions, camera movements, or poor camera angles. Within this one-hour segment, we analyzed the infant’s position and movements. A schematic overview of the analysis areas is shown in Figure 5. One sensor, located at position (8,5), was excluded from the analysis due to damage, which rendered its data unreliable.

To infer each infant’s posture and eventual out-of-bed moments, we examined the raw pressure signals, focusing on the distribution of pressure across the mat. Using the pressure distribution, we also calculated the center of mass and defined an area around it. This area was further divided into sub-areas corresponding to the infant’s limbs, enabling precise detection of limb movements (see Figure 5a).

To study the infants’ positions on the FM, we segmented the data into non-overlapping 10 s windows. For each window, we computed the median pressure value for each sensor. In addition, we analyzed the number of active sensors for each window. A sensor was considered active if it recorded a pressure greater than twice the standard deviation (2×std) for at least 80% of the window duration. In the end, an average number of active sensor per infant was retrieved, as well as the correlation of this average with the GA and the weight of the infants using Pearson’s correlation coefficient. To explore the feasibility of a simple and rapid classifier for posture recognition, features such as the average number of active sensors, GA, and weight of all infants were used in an extreme gradient boost classifier (XGB). It was chosen because of its ability in managing feature interactions and handling imbalanced data. Model performance was evaluated on this small dataset using Leave-One-Out Cross-Validation (LOOCV). This preliminary analysis aimed to identify non-lateral versus lateral positions, combining prone and supine positions into a single class due to the rarity of the prone position.

Subsequently, using the processing pipeline illustrated in Figure 5b, we quantified both the total movement and the percentage of movement in each sub-area over the entire one-hour segment, for which manual video annotations were available. To do so, from the median pressure distribution, the coordinates’ centers of mass (COMs) were found in each 10 s window in the 8 × 5 grid as follows:

$$row_{COM}=\frac{1}{P_{tot}}\sum_{i=1}^{8}\sum_{j=1}^{5}i*P_{i,j}\;col_{COM}=\frac{1}{P_{tot}}\sum_{i=1}^{8}\sum_{j=1}^{5}j*P_{i,j}$$

where $P_{tot}=\sum_{i=1}^{8}\sum_{j=1}^{5}P_{i,j}$ is the total pressure, and $P_{i,j}$ represents the pressure value at the grid cell in row i∈{1,…,8}, column j∈{1,…,5}, $row_{COM}\in \left\{1,\ldots ,8\right\}$, and $col_{COM}\in \left\{1,\ldots ,5\right\}$. Then, around the COM, an area of dimensions 6 × 3 was defined; this is the area where the infant lies, and it was used to examine the movement of the infant by analyzing the upper, lower, left, and right sections. To do so, the defined area was subdivided into four sub-areas, each of dimensions 3 × 2 (Figure 5a). Edge cases were also taken into account, as when the area around the COM exceeded the limits of the FM area. In these cases, a smaller area was defined around the COM, considering the limits of the FM area.

Then, the unprocessed signals from the selected sensors were filtered with three filters. Subsequently, a fourth-order Butterworth bandpass filter with a frequency range from 0.001 Hz to 0.4 Hz to extract motion-related frequencies, followed by a moving min–max using a window of 1 s with a moving step of one sample (0.02 s) to highlight movement’s fluctuations while keeping the same sample rate. Finally, a third-order Savitzky–Golay filter was used to mitigate fluctuations and minimize the likelihood that noisy peaks were misinterpreted as movements.

The filtered segments were then categorized by their respective sub-areas, and, for each sub-area in each 10 s segment, a score was determined as follows:

$$score_{sub-area}=\frac{\sum_{i=1}^{n}var_{sub-area}^{i}}{\sum_{j=1}^{m}var_{area}^{j}}$$

where $var_{sub-area}^{i}$ denotes the i-th variance within a sub-area, containing *n* sensors, and $var_{area}^{j}$ denotes the j-th variance within the area around the COM, containing *m* sensors. In this study, n=6 and m=15∨m=18, depending on the area being defined as an edge case or not, respectively.

After processing all segments, four normalized signals were obtained and subsequently binarized using a threshold set at the 95th percentile. For each non-overlapping 5 min window, the percentage of detected movements was computed from the binarized FM and MA signals per sub-area. The error for each window was then calculated as the difference between the movement percentages from FM and MA ($FM_{m\%}-MA_{m\%}$). Finally, the average error across all 5 min windows was computed using the following formula:

$$error_{avg}=\frac{1}{N}\sum_{i=1}^{N}\left(FM_{m\%}^{i}-MA_{m\%}^{i}\right)$$

where N is the number of 5 min windows. In the same way, the error of the still-period detection was calculated, where a still period is identified in the binarized FM signals as a 10 s segment where all four sub-areas detect a still period at the same time.

## 3. Results

### 3.1. Device Testing

**Figure 6:**
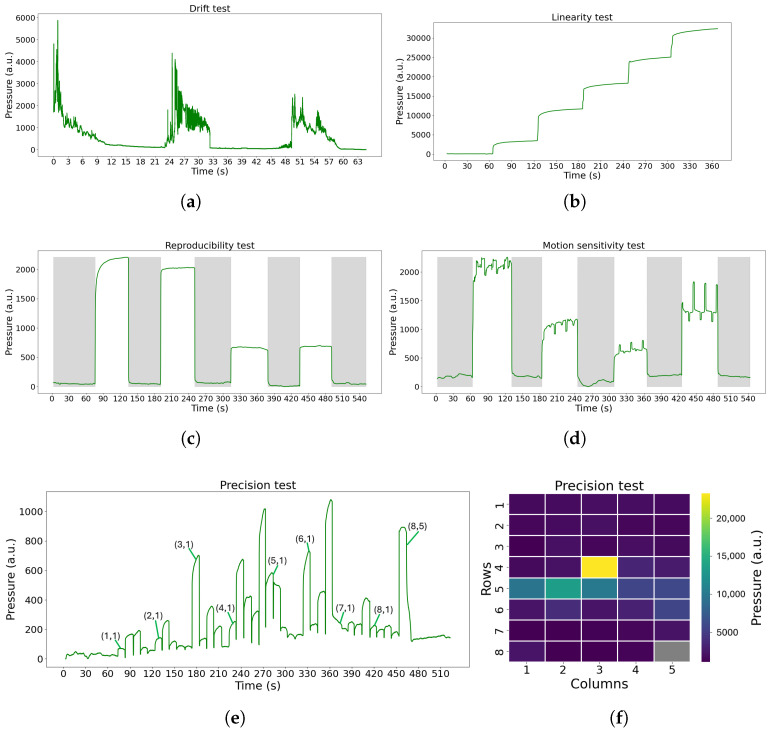
Results of the FM testing: (**a**) Drift test, 65 h. (**b**) Linearity test. (**c**) Reproducibility test: gray segments corresponds to periods when the Lego robot was not placed on the FM. (**d**) Motion sensitivity test: gray segments correspond to periods when the Lego robot was not placed on the FM. (**e**) Precision test (results per sensor over time): which sensor generated the corresponding pressure is indicated in the figure using the notation (row,column). (**f**) Precision test: FM graphical representation when node (4,3) was tested.

The results of the drift test are shown in Figure 6a. It can be seen that the system did have a significant drift during the test in three different moments, lasting around 8 h. These moments coincide with the daytime, when more light is present in the laboratory room where the test took place. Therefore, there might be influences of the environment in the readings when a high level of external light is present. The one-hour analysis revealed that the device has a median drift of −9.47%, while in a 24 h period, the same analysis revealed a median drift of −37.94%.

The results of the linearity test are shown in Figure 6b, as well as a linear response of the device; also, the steps are not of the same height. A per-sensor analysis revealed that occasionally some certain sensors tended to reach a state of saturation when subjected to an overall weight application of 5.7 kg or more.

The results of the reproducibility test are shown in Figure 6c. They show that repositioning has a large impact on the signal; with the last two times, the robot was positioned on the FM, having a significantly smaller amplitude compared to the first two. However, as we are mainly interested in motion analysis, shown in Figure 6d, it can be seen that even if reproducibility is not very robust, as the amplitude is lower, motion can still be detected.

The results of the precision test are shown in Figure 6e,f. Figure 6e illustrates how each sensor can register the pressure. Instead, in the heatmap in Figure 6f, each sensor is represented as a square in the map, and it can be seen that the nodes next to the node in testing, which in this case was the node in position (4,3), can be slightly influenced by the pressure change, since the FM material is also exerted. Also, a bit of crosstalk can be noticed for sensors in row 5, as they register some pressure when neighboring sensors are being tested. The information of this test can be further used for both electronic and digital signal calibration.

### 3.2. Clinical Study Data

#### 3.2.1. Position Monitoring and Out-of-Bed Moments

**Figure 7:**
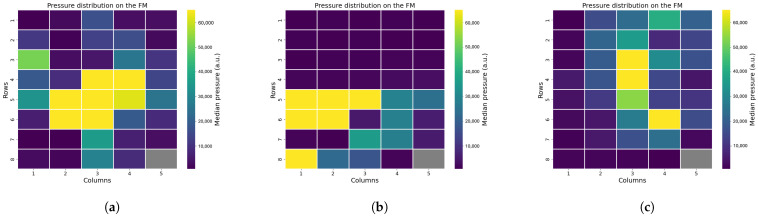
Position monitoring using the FM: (**a**) Infant in supine position. (**b**) Infant in prone position. (**c**) Infant on the side.

Among all 20 included preterm infants, for three of them, it was not possible to perform any annotation due to excessively dark video frames. From the analysis of the remaining 17 infants, it was possible to assess that, from the FM pressure distribution, the position can be inferred. The results of this analysis for three different infants are reported in Figure 7. In these cases, the infants were placed in a supine position (Figure 7a), prone position (Figure 7b), and on the side (Figure 7c). The differences in pressure distribution are noticeable, since in the supine or prone positions, a larger area can be identified compared to the lateral position.

**Figure 8:**
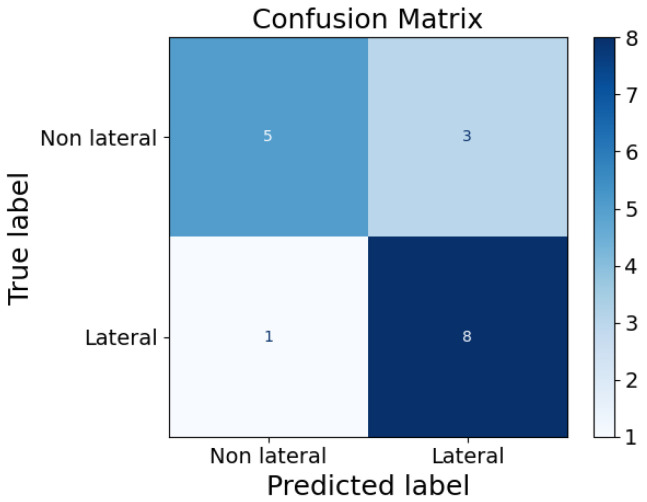
Confusion matrix of XGB classifier.

In addition, the average number of active sensors per infant was calculated (Table 1). Pearson correlation analysis demonstrated a strong positive association between the average number of active sensors and infant weight (r = 0.999, *p* < 0.001). A similarly strong, though slightly lower, correlation was observed between the average number of active sensors and gestational age (r = 0.911, *p* < 0.001). These findings indicate that both weight and gestational age are linearly related to the extent of sensor activation, with weight showing the strongest predictive relationship. Eventually, the XGB classifier achieved an accuracy of 76.47% in discriminating between non-lateral and lateral positions. In particular, the non-lateral position was more misclassified compared to the lateral postion, as shown in the confusion matrix in Figure 8. This might be due to the low number of features given to the classifier.

**Figure 9:**
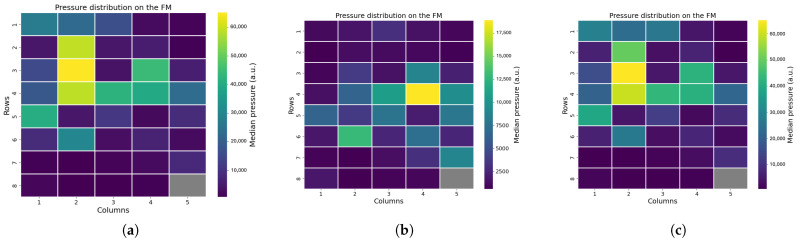
Out-of-bed moments: (**a**) Infant in bed. (**b**) Infant out of bed. (**c**) Infant in bed again.

In one measurement (infant 8), the infant was taken out of the bed for a while, as reported by the manual annotation of the video recording. The out-of-bed moment was clearly seen in the pressure distribution of the FM. In particular, three moments could be distinguished: in-bed, out-of-bed, and in-bed. These three moments are shown in Figure 9. It can be easily seen how the FM pressure distribution is considerably lower when the infant is not in the bed (Figure 9b), and then, as the infant is repositioned in the bed, the pressure distribution rises again gradually (Figure 9c). It is also seen that upon repositioning in bed, in a similar supine position, the pattern on the FM is also similar.

#### 3.2.2. Motion Monitoring

**Figure 10:**
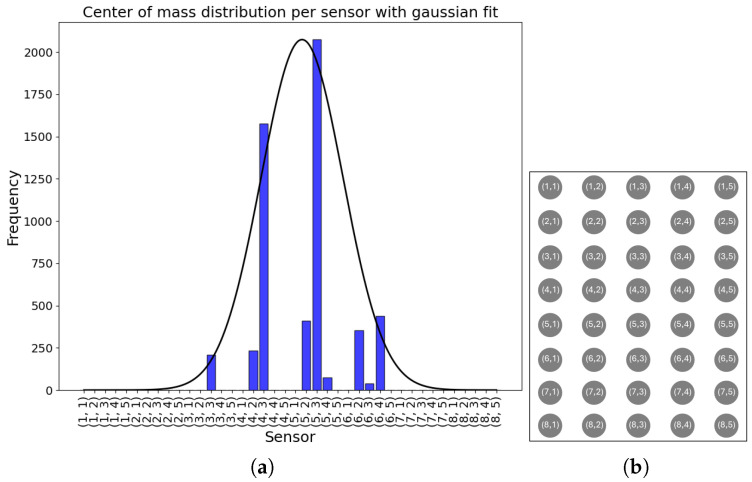
Center of mass distribution per sensor, all infants. (**a**) COM distribution with gaussian fit. (**b**) FM sensors schema.

In Figure 10, the results of the distribution of the COM detected for each 10 s segment for all infants are shown. It can be noticed that the sensors in position (5,3) and (4,3) were the most chosen as COM. Edge sensors in rows 1, 2, 7, and 8 and in columns 1 and 5 were never selected.

In Table 1, the error between the percentage of movement of the FM and the MAs in each sub-area is reported with some demographic information and the position of the infant. It can be seen that, in most cases, the error is below 10%, except for the left arm, which was the sub-area mostly miss-classified; in particular, the FM mostly underestimated the movement in this sub-area.

## 4. Discussion

In this study, we evaluated the feasibility of using a pressure-sensitive fiber-optic mattress (FM) for two-dimensional analysis of preterm infant movements and positioning. Alongside this, we developed a comprehensive and cost-effective testing protocol to assess smart mattresses prior to clinical use.

Before any medical device enters the clinical setting, particularly NICUs and NMCUs where vulnerable preterm infants are cared for, assessment for both safety and functionality is warranted to ensure consistent performance and reliable detection of physiological indicators such as movement. However, emerging technologies like smart mattresses, camera-based monitors, and wearable sensors often require new testing protocols, as conventional methods may not suit their measurement principles. Standardized frameworks for these devices remain scarce, and developing testing methods can be costly and complex, and, although several studies have explored other types of mattress-embedded sensors [21,22,23], most of the existing literature generally omits detailed discussions of potential bench-test methodologies, crucial for performance assessment and quality assurance purposes. To address this gap, we proposed a simple, replicable, and affordable testing protocol designed to evaluate motion detection and key features in pressure-sensitive mattresses, aiming to support faster, high-quality device validation.

Applying this protocol to the FM prototype allowed us to identify technical issues before starting the clinical study, enabling early improvements. First, the drift test showed that the FM was highly sensitive to changes in ambient light, suggesting that enhanced shielding is necessary to minimize this effect. Then, with the linearity test, we were able to assess a small non-linear behavior of the device, which might influence the small movements detection. With the use of a Lego Mindstorm robot, we were also able to mimic an infant laying on the FM, which allowed us to assess the device’s ability to sense movements, even if it lacked a bit in reproducibility. Eventually, in the precision test, instances of mechanical crosstalk were observed, where adjacent sensors unintentionally detected pressure applied to neighboring sensors. In future prototypes of the FM, different materials of the POF crossing can be tested to attenuate the crosstalk; moreover, signal processing techniques can be applied to already collected signals to better isolate useful information. We mainly aim at redesign the device, while signal-processing techniques can be applied to further mitigate the problem. Additionally, sensors in position (8,5) exhibited manufacturing defects, consistently reporting elevated values regardless of pressure, while sensors in row 5 produced false readings even when no object was present. In general, the testing phase also revealed variability in sensor sensitivity, indicating the need for recalibration before data analysis.

A limitation to our protocol is that it was tested in a laboratory environment, which may not fully reflect the conditions of a clinical setting. First, the FM was not tested with an incubator used in the NICU, which could cause additional edge effects not yet considered. In addition, the lighting and noise present in a real NICU setting could differ from that in the laboratory. However, as this will vary from NICU to NICU, in the test report, the light conditions during the test should be mentioned. It can be valuable to test in different light conditions. However, given its simplicity and flexibility, the protocol can be easily adapted for use in environments such as the NICU, allowing for real-world validation.

The second part of this study focused on the analysis of clinical data. The presented analysis demonstrated the feasibility of using the FM in a two-dimensional motion detection and position monitoring in preterm infants.

Position analysis confirmed that it is possible to reliably differentiate between supine, prone, and lateral positions based on pressure distribution patterns analysis. Supine and prone positions produced broader areas of sensor activation, while lateral positions showed active sensors concentrated along a line. Moreover, the pressure distribution changes with the growth of the infant, as older infants demonstrated larger sensor activation compared to younger ones. We also included an easy and fast XGB classifier, which demonstrated elevated accuracy in distinguish between non-lateral and lateral positions. Additional analysis of the pressure distribution demonstrated that instances where the infant was out of bed were detectable are both clinically important and useful as recalibration points during data analysis. In daily clinical care, monitoring these aspects can be important, as infant positioning is associated with various clinical conditions, such as feeding tolerance and cardiorespiratory status [24]. Moreover, frequent repositioning plays a key role in supporting the infant’s development and maintaining healthy skin integrity [25,26]. In addition, monitoring patient handling and out-of-bed moments is crucial for artifact reduction and alarm management [27,28].

In general, in this study, position analysis is restricted by edge effects that can modify real readings, including those caused by bed sheets or other materials on the mattress, which alter the pressure distribution. Therefore, further refinements are needed to enhance the position analysis using the FM. Regarding the XGB classifier, its performance needs to be evaluated on a larger dataset, edge effects need to be taken into consideration to enhance its accuracy, and more and different features can be used to obtain better classification. Also, other classifiers can be tested, and their performances can be compared. In addition, the prone position should also be added to the classifier to make it complete, but it was not represented enough in this dataset, as most preterm infants at the end of the NICU stay are laid in a supine or lateral position.

Automating the monitoring of the infant position offers significant clinical benefits. Positioning affects key health factors such as respiratory function, oxygenation, lung compliance, apnea events, and feeding tolerance [3,4,5]. It may also provide clues to neurological abnormalities, although understanding these would require more detailed information than just the infant’s posture in the incubator or bed. Additionally, automated position tracking can support the development of more advanced motion detection systems in the future.

In the motion analysis, we calculated the center of mass (COM) of the pressure distribution every 10 s to estimate the infant’s location and movement. The algorithm generally located the COM within the expected central region of the FM; however, edge effects occasionally influenced the results, such as when pressure from the tucked bedsheets activated side sensors. This affected both COM calculation and motion detection accuracy, highlighting the need for improved handling of edge-related artifacts in future analyses.

The FM effectively detected movements of the infant limb, although some limitations emerged. Movements made in lateral positions, particularly arms not in contact with the mattress, were sometimes undetected by FM, even when visible on camera, as, in this case, no pressure was applied on the FM. In contrast, certain leg movements were difficult to verify by camera when covered with bedsheets or when the camera angle was suboptimal. However, the FM achieved a low average error, even in lateral positions, and demonstrated the ability to detect small limb movements as accurately as camera-based systems. The combination of the FM and a camera system could be useful for a more robust limb motion detection, while overcoming the limitations derived from both systems.

The motion analysis was limited by the simplified model of the limbs, which have a complex geometric shape. However, due to the design of the FM sensors, it was not possible to obtain a more reliable and accurate representation of the limbs than the one presented. Future FM design could overcome this limitation by providing more pressure points, from which a more complex shape of the limbs can be modeled. A second limit can be found in the small sample size, which allowed us to perform a first feasibility study. In the future, a longer study is needed to address the long-term monitoring ability of the FM. A third limitation is found in the signals’ alignment, which was performed manually, as is typically performed for this first prototype stage of sensor development. The signals’ synchronization can be improved in future studies with an automated method.

In contrast to existing technologies that depend on smart mattresses [14,15], our method provides a simpler and more efficient approach to analyze infant motion patterns in a two-dimensional space. Although further research is required to support real-time application, the use of machine learning techniques offers a promising direction. Additionally, by achieving motion detection performance comparable to camera-based systems, our solution delivers a precise, non-obtrusive alternative with a more streamlined and dependable analysis process.

In general, this study shows that the proposed testing protocol is a practical and valuable tool to identify technical issues and evaluate motion detection capabilities in smart mattresses. Moreover, the FM was proven feasible for monitoring infant position and movements. However, further refinements, including improved calibration, light shielding, and handling of edge effects, are needed to optimize its performance for clinical use.

## 5. Conclusions

This study introduced a pressure-sensitive fiber-optic mattress, entirely made of plastic material, and a simple and cost-effective testing protocol to evaluate smart mattresses prior to clinical use. The protocol effectively identified technical issues, including mechanical crosstalk, sensor defects, and environmental sensitivity, allowing for early improvements. Clinical data analysis confirmed the feasibility of using the FM for monitoring infant position and movements, reliably distinguishing between supine, prone, and lateral positions, and detecting small limb motions more accurately than camera-based systems alone.

Although the results are promising, limitations such as light sensitivity, edge effects, and undetected movements highlight areas for refinement. Future work should focus on improving calibration, environmental shielding, and real-world validation in clinical environments such as NICUs to confirm long-term reliability and clinical utility.

## Figures and Tables

**Table 1:** Average error between FM and MAs in motion detection per limbs and still periods. Green = |x| < 10%, orange = 10%≤|x|<30%, and red = |x|≥30%. GA = gestational age, Avg. = Average, Err. = Error, LL= Left leg, RL= Right Leg, LA = Left Arm, RA = Right Arm. Values for infants 9, 15, and 19 are missing, as no manual annotations could be performed.

**Infant**	**GA** ** (Weeks)**	**Weight** ** (g)**	**Position**	**Avg. Active** ** Sensors**	Avg. Err. per Limb				**Avg. Err.** ** per Still**
					**LL**	**RL**	**LA**	**RA**	
**1**	31.6	1560	Supine	3.23	6.67%	0.00%	32.86%	−9.57%	−33.14%
**2**	27.3	1440	Lateral	4.81	6.67%	0.00%	0.00%	3.33%	0.10%
**3**	30.7	1990	Supine	9.11	−0.67%	0.00%	−25.67%	−39.00%	15.00%
**4**	36.1	1810	Lateral	5.71	0.00%	0.00%	13.33%	−6.79%	−23.15%
**5**	33.6	1985	Supine	3.75	−43.85%	−39.23%	−27.44%	−19.74%	47.61%
**6**	32.7	2190	Lateral	8.36	−7.27%	0.00%	−13.38%	5.00%	−7.50%
**7**	33.1	2230	Lateral	12.00	6.67%	3.33%	−15.28%	−0.83%	10.83%
**8**	33.1	2310	Supine	5.11	0.00%	3.33%	−2.00%	0.00%	−0.33%
**10**	32.3	1150	Lateral	9.91	3.33%	2.50%	−29.20%	−11.94%	13.86%
**11**	27.1	2500	Prone	6.19	0.00%	0.00%	−20.83%	1.39%	−9.79%
**12**	27.1	2725	Supine	8.91	0.00%	0.00%	−1.67%	−43.33%	−4.72%
**13**	25.3	1380	Lateral	9.88	26.67%	−0.74%	0.00%	−6.30%	−25.56%
**14**	26.0	1830	Supine	8.50	−1.82%	−1.82%	−35.45%	0.61%	−3.03%
**16**	28.7	1230	Supine	10.88	3.33%	0.00%	0.00%	0.00%	9.63%
**17**	28.7	1100	Lateral	14.89	0.00%	26.67%	0.00%	0.00%	−20.33%
**18**	30.3	1670	Lateral	15.36	29.10%	0.00%	−41.60%	−28.48%	−7.91%
**20**	29.5	1395	Lateral	13.30	0.00%	0.00%	−58.35%	3.38%	19.74%
**Median**	**30.3**	**1810**	**/**	**8.91**	**0.00%**	**0.00%**	**−13.38%**	**−0.83%**	**−3.03%**

## Data Availability

The data are not publicly available due to privacy reasons.

## Abbreviations

The following abbreviations are used in this manuscript:

AUC	Area under the curve
CI	Chest impedance
COM	Center of mass
DAC	Data acquisition and control
ECG	Electrocardiography
EMFi	Electromechanical film sensor
FM	Fiber-optic pressure-sensitive mattress
LED	Light emitting diode
LOOCV	Leave-one-out cross-validation
MA	Manual annotations of video recordings
NICU	Neonatal intensive care unit
NMCU	Neonatal medium care unit
POF	Polymer optical fiber
XGB	Extreme gradient boost
